# Estimated Community Seroprevalence of SARS-CoV-2 Antibodies — Two Georgia Counties, April 28–May 3, 2020

**DOI:** 10.15585/mmwr.mm6929e2

**Published:** 2020-07-24

**Authors:** Holly M. Biggs, Jennifer B. Harris, Lucy Breakwell, F. Scott Dahlgren, Glen R. Abedi, Christine M. Szablewski, Jan Drobeniuc, Nirma D. Bustamante, Olivia Almendares, Amy H. Schnall, Zunera Gilani, Tiffany Smith, Laura Gieraltowski, Jeffrey A. Johnson, Kristina L. Bajema, Kelsey McDavid, Ilana J. Schafer, Vickie Sullivan, Lili Punkova, Alexandra Tejada-Strop, Raiza Amiling, Claire P. Mattison, Margaret M. Cortese, S. Elizabeth Ford, Lynn A. Paxton, Cherie Drenzek, Jacqueline E. Tate, CDC Field Surveyor Team, CDC Field Surveyor Team, Nicole Brown, Karen T. Chang, Nicholas P. Deputy, Rodel Desamu-Thorpe, Chase Gorishek, Arianna Hanchey, Michael Melgar, Benjamin P. Monroe, Carrie F. Nielsen, Gerald J. Pellegrini, Mays Shamout, Laura I. Tison, Sara Vagi, Rachael Zacks

**Affiliations:** ^1^CDC COVID-19 Response Team; ^2^Georgia Department of Public Health; ^3^Epidemic Intelligence Service, CDC; ^4^DeKalb County Board of Health, Decatur, Georgia; ^5^Fulton County Board of Health, Atlanta, Georgia.; CDC COVID-19 Response Team; CDC COVID-19 Response Team; CDC COVID-19 Response Team; CDC COVID-19 Response Team; CDC COVID-19 Response Team; CDC COVID-19 Response Team; CDC COVID-19 Response Team; CDC COVID-19 Response Team; CDC COVID-19 Response Team; CDC COVID-19; Response Team; CDC COVID-; CDC COVID-; CDC COVID-19 Response Team; CDC COVID-19 Response Team.; Response Team; Response Team

Transmission of SARS-CoV-2, the virus that causes coronavirus disease 2019 (COVID-19), is ongoing in many communities throughout the United States. Although case-based and syndromic surveillance are critical for monitoring the pandemic, these systems rely on persons obtaining testing or reporting a COVID-19–like illness. Using serologic tests to detect the presence of SARS-CoV-2 antibodies is an adjunctive strategy that estimates the prevalence of past infection in a population. During April 28–May 3, 2020, coinciding with the end of a statewide shelter-in-place order, CDC and the Georgia Department of Public Health conducted a serologic survey in DeKalb and Fulton counties in metropolitan Atlanta to estimate SARS-CoV-2 seroprevalence in the population. A two-stage cluster sampling design was used to randomly select 30 census blocks in each county, with a target of seven participating households per census block. Weighted estimates were calculated to account for the probability of selection and adjusted for age group, sex, and race/ethnicity. A total of 394 households and 696 persons participated and had a serology result; 19 (2.7%) of 696 persons had SARS-CoV-2 antibodies detected. The estimated weighted seroprevalence across these two metropolitan Atlanta counties was 2.5% (95% confidence interval [CI] = 1.4–4.5). Non-Hispanic black participants more commonly had SARS-CoV-2 antibodies than did participants of other racial/ethnic groups (p<0.01). Among persons with SARS-CoV-2 antibodies, 13 (weighted % = 49.9; 95% CI = 24.4–75.5) reported a COVID-19–compatible illness,* six (weighted % = 28.2; 95% CI = 11.9–53.3) sought medical care for a COVID-19–compatible illness, and five (weighted % = 15.7; 95% CI = 5.1–39.4) had been tested for SARS-CoV-2 infection, demonstrating that many of these infections would not have been identified through case-based or syndromic surveillance. The relatively low seroprevalence estimate in this report indicates that most persons in the catchment area had not been infected with SARS-CoV-2 at the time of the survey. Continued preventive measures, including social distancing, consistent and correct use of face coverings, and hand hygiene, remain critical in controlling community spread of SARS-CoV-2.

DeKalb and Fulton counties had the highest numbers of reported COVID-19 cases among Georgia counties at the time of survey initiation (approximately 1,900 and 2,700, respectively). A two-stage cluster sampling design, stratified by county, was used to target a representative sample of 420 households.^†^ Within each county, 30 census blocks were randomly selected with probability proportional to number of occupied households (per 2010 U.S. Census) without replacement. Selection of the census blocks was performed using the Community Assessment for Public Health Emergency Response Geographic Information System Toolbox.^§^ Within each census block, systematic sampling was used to select seven households for participation; a centroid starting location was defined and every n^th^ household (defined as number of households in the cluster divided by seven) was approached for participation.

The survey was conducted during April 28–May 3, overlapping partially with the Georgia shelter-in-place order for all residents (April 3–30). A household was defined as a living space shared by one or more persons, excluding correctional facilities, long-term care facilities, dormitories, or other institutional settings. Unoccupied buildings were excluded. If a household declined participation, did not respond to an initial door knock, or could not be enrolled for another reason,^¶^ an adjacent household was selected. All household members who spent an average of ≥2 nights per week in the home were invited to participate. A blood sample for serology was required from at least one household member for household enrollment. A standardized questionnaire was administered to participants, assessing household and demographic characteristics, chronic medical conditions, recent illnesses and associated symptoms, previous testing for SARS-CoV-2, and potential exposures.

This investigation was determined by CDC and the Georgia Department of Public Health to be public health surveillance.** Participants or their parent or guardian provided written consent. Individual test results were returned to participants who indicated that they would like to receive them. After the survey was completed, CDC and the Georgia Department of Public Health participated in a community outreach event to address community questions and concerns about the survey.

Phlebotomists used standard venipuncture technique to collect blood in households from consenting participants. Blood was collected in K2-EDTA tubes and transported to a CDC laboratory certified under the Clinical Laboratory Improvement Amendments of 1988 (CLIA), where plasma was separated into aliquots in Nalgene cryogenic vials. One aliquot was heat-treated at 56°C (132.8°F) for 10 minutes, and then tested using the qualitative VITROS anti-SARS-CoV-2 total antibody in vitro diagnostic test on the automated VITROS 3600 Immunodiagnostic System (Ortho Clinical Diagnostics)^††^ Verification of the assay performance characteristics was performed by the CDC testing laboratory (sensitivity = 93.2%, specificity = 99.0%, accuracy = 96.8%, reproducibility = 100.0%, and serum/plasma equivalency = 95.6%).

The age, sex, and racial/ethnic distributions of participants were compared with those of the catchment area population using one-way chi-squared goodness-of-fit tests. Initial weights were computed as the inverse of the probability of selection and adjusted using a raking algorithm so that the marginal distribution of age group, sex, and race/ethnicity of the sample closely agreed with population estimates from the U.S. Census Bureau (*1*,*2*). Crude values and population estimates (weighted proportions) are reported for describing the survey participants. Characteristics of participants with (seropositive) and without (seronegative) presence of SARS-CoV-2 antibodies were compared using a score test for independence that performs well even with sparse data (*3*). Wilson’s interval was used for computing 95% CIs (*4*,*5*). Analysis was conducted using SAS (version 9.4; SAS Institute).

Among 1,675 households approached, 397 (23.7%) were enrolled, attaining 94.5% of the targeted 420 households.^§§^ All 60 census blocks were represented, with an average of 6.6 (range = 2–7) households enrolled per census block. Participating households had a total of 1,122 household members (median household size = two; range = 1–11); 708 persons provided a blood sample for serology, and 696 (98.3%) persons from 394 (99.2%) households had a serology result.^¶¶^ Compared with census data for the counties, participants were less frequently children aged <18 years and more likely to be non-Hispanic white (Table 1).

**TABLE 1:** Unweighted demographic characteristics of survey participants with a SARS-CoV-2 serology test result, compared with 2018 postcensal estimates for the overall catchment area — DeKalb and Fulton counties, Georgia, April 28–May 3, 2020

Characteristic	No. (%)		p value^†^
	Participants (N = 696)	Catchment area* (N = 1,806,672)	
**Gender**			0.241
Male	317 (45.6)	866,297 (47.9)	
Female	377 (54.2)	940,375 (52.1)	
Other^§^	2 (0.3)	0 (—)	
**Age group (yrs)**			<0.001
0–17	48 (6.9)	404,349 (22.4)	
18–49	347 (49.9)	860,956 (47.6)	
50–64	189 (27.2)	324,517 (18.0)	
≥65	112 (16.1)	216,850 (12.0)	
**Race/Ethnicity**			<0.001
White, non-Hispanic	329 (47.3)	634,436 (35.1)	
Black, non-Hispanic	266 (38.2)	854,544 (47.3)	
Hispanic	44 (6.3)	141,394 (7.8)	
Asian/Pacific Islander, non-Hispanic	29 (4.2)	128,981 (7.1)	
Multiple race/Other/Unknown	28 (4.0)	47,317 (2.6)	

**Source:** National Center for Health Statistics. Vintage 2018 postcensal estimates. https://www.cdc.gov/nchs/nvss/bridged_race/data_documentation.htm#Vintage2018.

* DeKalb County and Fulton County combined; 2018 postcensal estimates.

^†^ One-way chi-squared goodness-of-fit tests comparing sample with catchment area demographics.

^§^ Excluded when testing against the distribution of the catchment area.

Overall, 19 (2.7%) of 696 participants, representing 15 (3.8%) of 394 households in 14 census blocks, were seropositive. The weighted seroprevalence in the total catchment area was 2.5% (95% CI = 1.4–4.5). Among age groups, seroprevalence estimates were highest among adults aged 18–64 years; no children were seropositive (Table 2). Among racial/ethnic groups, the highest estimated seroprevalence (5.2%; 95% CI = 2.9–9.1) was among non-Hispanic black participants, which was significantly higher than that among all other racial/ethnic groups combined (p<0.01).

**TABLE 2:** Demographic characteristics of participants with and without SARS-CoV-2 antibodies and estimated seroprevalence — DeKalb and Fulton counties, Georgia, April 28–May 3, 2020

Characteristic	Participants with SARS-CoV-2 antibodies (N = 19)		Participants without SARS-CoV-2 antibodies (N = 677)		Estimated seroprevalence (95% CI)
	No.	Weighted proportion,* % (95% CI)	No.	Weighted proportion,* % (95% CI)	
Total	19	**100**	**677**	**100**	**2.5 (1.4–4.5)**
**Sex**					
Male	8	50.1 (25.6–74.7)	309	47.8 (43.3–52.2)	2.6 (1.1–6.3)
Female	11	49.9 (25.3–74.4)	366	52.0 (47.6–56.5)	2.4 (1.1–5.1)
Other	0	0 (—)	2	0.2 (0.0–0.9)	—
**Age group (yrs)**					
0–17	0	0 (—)	48	22.8 (16.7–30.3)	—
18–49	12	61.6 (35.2–82.6)	335	47.4 (40.8–54.1)	3.3 (1.6–6.4)
50–64	6	35.2 (14.8–62.8)	183	17.5 (14.5–21.1)	4.9 (1.8–12.9)
≥65	1	3.2 (0.4–21.8)	111	12.3 (9.4–15.8)	0.7 (0.1–4.5)
**Race/Ethnicity**					
White, non-Hispanic	2	4.6 (0.7–23.7)	327	37.2 (27.8–47.7)	0.3 (0.1–1.7)
Black, non-Hispanic	16	93.5 (73.8–98.7)	250	44.2 (33.8–55.1)	5.2 (2.9–9.1)
Hispanic	0	0 (—)	44	7.7 (4.2–13.5)	—
Asian/Pacific Islander, non-Hispanic	0	0 (—)	29	6.9 (2.5–17.6)	—
Multiple race/Other/Unknown	1	1.9 (0.2–19.8)	27	4.0 (2.1–7.5)	1.2 (0.1–14.1)

**Abbreviation:** CI = confidence interval.

* Weights were computed as the inverse of the probability of selection and adjusted so that the marginal distribution of age group, sex, and race/ethnicity of the sample closely agreed with population estimates; presented as column percentages.

Two participants from separate households reported a previously confirmed SARS-CoV-2 infection; both were seropositive (Table 3). A COVID-19–compatible illness during 2020 was reported by 229 (weighted % = 33.3; 95% CI = 27.6–39.6) *** seronegative participants and 13 (weighted % = 49.9; 95% CI = 24.4–75.5) seropositive participants (p = 0.31). Among seropositive persons, none had been hospitalized, six (weighted % = 28.2; 95% CI = 11.9–53.3) had sought medical care for a COVID-19–compatible illness, and five (weighted % = 15.7; 95% CI = 5.1–39.4) had been previously tested for SARS-CoV-2 infection.

**TABLE 3:** Characteristics and exposures of participants with and without SARS-CoV-2 antibodies — DeKalb and Fulton counties, Georgia, April 28–May 3, 2020

Characteristic	Participants with SARS-CoV-2 antibodies (N = 19)		Participants without SARS-CoV-2 antibodies (N = 671)*	
	No.	Weighted proportion,^†^ % (95% CI)	No.	Weighted proportion,^†^ % (95% CI)
**Illness history during 2020**				
COVID-19–compatible illness^§^	13	49.9 (24.4–75.5)	229	33.3 (27.6–39.6)
Any illness with cough or shortness of breath	10	31.1 (13.8–55.9)	188	26.2 (21.2–32.0)
Any illness with fever/feeling feverish	12	47.9 (23.3–73.6)	147	21.7 (16.7–27.6)
Any illness with loss of taste or smell	8	28.4 (12.4–52.7)	38	8.2 (4.9–13.5)
Sought medical care for illness^¶^	6	28.2 (11.9–53.3)	117	16.3 (12.1–21.6)
Hospitalized because of illness	0	0 (—)	5	0.9 (0.4–2.2)
Missed work or school because of illness	10	42.4 (20.1–68.2)	121	19.7 (15.1–25.4)
**Previous test for SARS-CoV-2**				
None	14	84.3 (60.6–94.9)	643	97.1 (95.4–98.2)
Positive result	2	7.0 (1.5–27.0)	0	0 (—)
Negative result	1	4.4 (0.7–23.5)	23	2.6 (1.6–4.3)
Unknown result**	2	4.3 (0.7–23.3)	5	0.3 (0.1–1.1)
**Medical history**				
Any chronic condition^††^	7	20.3 (8.1–42.5)	309	39.8 (34.0–45.8)
Chronic lung disease	1	1.5 (0.1–19.2)	86	14.0 (10.8–18.0)
Cardiovascular disease	5	15.5 (5.4–37.2)	167	18.5 (14.9–22.7)
Chronic kidney disease	0	0 (—)	8	1.1 (0.4–3.0)
Liver disease	0	0 (—)	8	0.6 (0.2–1.5)
Diabetes mellitus^§§^	2	5.3 (0.9–24.6)	61	7.2 (5.2–10.0)
Autoimmune/Rheumatologic condition	2	5.9 (1.2–25.6)	27	2.8 (1.8–4.3)
Immunocompromising condition or therapy	0	0 (—)	46	5.1 (3.6–7.2)
Neurologic condition	0	0 (—)	18	2.8 (1.7–4.7)
Seasonal allergies	10	43.3 (21.8–67.7)	404	59.7 (52.7–66.3)
Pregnant or postpartum^¶¶^	0	0 (—)	9	1.4 (0.5–3.5)
**Known exposures to ill persons**				
Contact with ≥1 person with confirmed COVID-19	2	7.8 (1.8–28.0)	30	6.5 (3.8–10.9)
Cared for person with confirmed COVID-19	2	7.8 (1.8–28.0)	12	2.5 (1.2–5.3)
Contact with ≥1 person with respiratory symptoms (not known confirmed COVID-19)	5	20.9 (7.3–46.9)	139	21.9 (17.3–27.2)
**Travel during 2020**				
International travel (outside of the United States)	2	9.8 (2.6–30.5)	81	11.1 (7.2–16.7)
Domestic travel (outside of Georgia)	4	24.3 (9.2–50.5)	254	32.4 (26.7–38.8)
**Work setting**				
Attend or work in a school or daycare***	6	21.7 (8.9–44.1)	188	38.8 (31.3–47.0)
Work in a health care setting***	5	19.9 (7.2–44.6)	56	8.4 (5.3–13.1)
Outpatient or urgent care clinic	3	10.0 (2.4–33.3)	17	2.1 (1.2–3.8)
Hospital or emergency department	2	10.0 (2.7–30.9)	13	1.3 (0.6–2.4)
Long-term care or assisted living facility	0	0 (—)	3	0.9 (0.2–3.3)
>1 setting	0	0 (—)	4	0.4 (0.1–1.2)
Other^†††^	0	0 (—)	19	3.8 (1.9–7.5)
**Work industry (participants aged ≥18 years)** ^§§§^				
Utilities/Construction/Manufacturing	0	0 (—)	42	4.7 (3.2–6.7)
Warehouse/Shipping/Parcel delivery	2	19.6 (5.2–52.0)	9	0.8 (0.4–1.8)
Restaurants/Bars/Food services/Accommodation	1	10.7 (2.1–39.9)	23	3.4 (2.1–5.4)
Retail/Grocery stores	0	0 (—)	19	2.0 (1.2–3.4)
Transportation	0	0 (—)	14	1.5 (0.8–2.7)
Education/Child day care	0	0 (—)	48	6.3 (4.6–8.6)
Health care^¶¶¶^	6	37.6 (15.6–66.1)	53	7.4 (4.7–11.4)
Barber shop/Beauty salon/Personal services	1	3.9 (0.6–22.8)	9	1.0 (0.5–2.1)
Finance/Banking/Insurance and real estate/Rental/Leasing	0	0 (—)	34	3.8 (2.6–5.6)
Professional/Scientific/Technical services	0	0 (—)	47	7.1 (4.5–11.0)
Public administration	2	4.7 (0.8–23.9)	22	2.5 (1.5–4.1)
Religious organizations	1	2.9 (0.3–21.4)	5	0.3 (0.1–1.1)
Student	2	5.0 (0.9–24.3)	14	1.6 (0.9–2.9)
Other industry	0	0 (—)	53	6.4 (4.6–8.7)
Retired or unemployed	3	7.5 (1.7–27.6)	154	18.8 (14.7–23.8)
Insufficient information to classify	1	8.0 (1.6–32.6)	78	9.6 (6.7–13.5)
**Dwelling type**				
Single unit (including townhouses)	13	48.0 (23.5–73.5)	489	71.9 (59.4–81.7)
Multiunit (≥2 housing units per building)	6	52.0 (26.5–76.5)	175	27.2 (17.5–39.7)

**Abbreviations:** CI = confidence interval; COVID-19 = coronavirus disease 2019; CSTE = Council of State and Territorial Epidemiologists.

* Denominator = six of the 677 seronegative participants had missing data.

^†^ Weights were computed as the inverse of the probability of selection and adjusted so that the marginal distribution of age group, sex, and race/ethnicity of the sample closely agreed with population estimates; column percentages are presented.

^§^ Based on clinical criteria in the CSTE COVID-19 case definition. (https://cdn.ymaws.com/www.cste.org/resource/resmgr/2020ps/interim-20-id-01_covid-19.pdf.

^¶^ Went to a doctor, clinic, emergency department, saw a doctor remotely through telemedicine because of the illness, or was hospitalized overnight for the illness.

** Includes test result still pending at the time of the survey.

^††^ Some persons reported more than one chronic condition; chronic conditions included chronic lung disease, cardiovascular diseases, chronic kidney disease, liver disease, diabetes mellitus, autoimmune or rheumatologic condition, immunocompromising condition or therapy, and neurologic condition.

^§§^ Includes reports of prediabetes.

^¶¶^ Postpartum defined as up to 6 weeks after childbirth.

*** Since January 2020 but not necessarily at the time of the survey.

^†††^ Additional settings reported included functional medicine, physical therapy clinic, support office/building, mental health clinic, research administration, emergency medical technician, plasma donation center, home health care, federal OSHA clinic, research clinic, volunteer at a hospital, technician-phone interviews, dietician office, school nurse, dentist office, community clinic, and pharmaceutical representative.

^§§§^ Work information collected in a free text field was coded based on the Census Industry and Occupation Classification System. The codes were then combined into broad industry categories based on National Health Interview Survey simple and detailed recode categories. https://www.cdc.gov/niosh/topics/coding/analyze.html.

^¶¶¶^ One seropositive participant worked in health care but not in a health care setting (reported full-time telework in 2020).

Among seropositive participants, two had known contact with a person with COVID-19. Work in a health care setting, although not necessarily as a direct care provider, was reported by five (weighted % = 19.9; 95% CI = 7.2–44.6) seropositive participants, and 56 (weighted % = 8.4; 95% CI = 5.3–13.1) seronegative participants (p = 0.28). Living in a multi-unit dwelling (two or more units per building) was reported for six (weighted % = 52.0; 95% CI = 26.5–76.5) seropositive participants and 175 (weighted % = 27.2; 95% CI = 17.5–39.7) seronegative participants (p = 0.20).

## Discussion

A door-to-door household survey conducted in two counties in metropolitan Atlanta during April 28–May 3, 2020, found an estimated 2.5% seroprevalence of SARS-CoV-2 antibodies. This suggests that most of the population had not been infected with SARS-CoV-2 at the time of the survey, which occurred at the end of the statewide shelter-in-place order. Few U.S. studies are available for comparison; those available used different methods and estimated seroprevalence during April at 1.8% in Boise, Idaho; 4.7% in Los Angeles, California; and 14.0% in New York (including New York City) (*6*–*8*).

In this metropolitan Atlanta survey, an estimated one half of seropositive persons recalled having had a COVID-19–compatible illness, approximately one third sought medical care for the illness, and even fewer had a test for SARS-CoV-2 infection. These findings highlight that many SARS-CoV-2 infections would have been missed by case-based surveillance, which requires receiving medical care in the health care system or a test for SARS-CoV-2, and by syndromic surveillance, which relies on symptomatic illness. As testing practices change during the course of the pandemic, this pattern, reflecting findings at the end of April, might also change.

SARS-CoV-2 seropositivity was associated with non-Hispanic black race/ethnicity in this survey. Although the number of seropositive persons in the survey are small for assessing differences between seronegative and seropositive persons, this finding is congruent with other data indicating that non-Hispanic blacks have been disproportionally affected by the COVID-19 pandemic (*9*). A multitude of factors might play a role in this disparity (e.g., social determinants of health, including factors related to housing, economic stability, and work circumstances). In general, black persons have increased likelihood of exposure through work in frontline industries and are more likely to live in housing structures with higher population density (*10*).

Many aspects of the immune response to SARS-CoV-2 infection are unknown. Understanding rates of seroconversion among asymptomatic persons, the duration of detectable circulating antibodies in relation to illness severity, and the potential impact of host factors (e.g., age and underlying medical conditions) on seroconversion are essential for interpreting SARS-CoV-2 seroprevalence data. It is also unknown whether antibodies, as detected by commonly available serologic assays, confer immunity, a critical factor in understanding the implications of seroprevalence estimates.

The findings in this report are subject to at least six limitations. First, the sampling frame was derived from 2010 census data and did not reflect subsequent changes in housing and occupancy. Second, participation was voluntary, and the overall participation rate of approached households was low. The effect of nonresponse bias on the seroprevalence estimates is unknown; many factors might have influenced a person’s willingness to participate, including the likelihood of being at home during the shelter-in-place order, mistrust of a door-to-door survey among community members, and the probability that the person was seropositive, all of which might affect the survey’s representativeness. Active community engagement beginning at the design of the survey is an important component to gain trust and potentially improve participation. Third, racial and ethnic minority populations and children aged <18 years were underrepresented; the lack of seropositivity among persons aged <18 years might have biased the final seroprevalence estimate toward zero. Fourth, the survey was powered to determine an overall seroprevalence estimate and not for subgroup analyses. The number of seropositive participants was low, resulting in wide CIs for weighted proportions. Fifth, all serologic assays have associated error that can result in false-positive or false-negative results. Particularly, false-positive results are of concern when the overall population seroprevalence is low. The accuracy and precision of the final seroprevalence estimate is affected by both test and sampling error. Finally, case numbers in the Georgia counties where this survey was conducted have increased substantially since the survey was conducted; therefore, the seroprevalence reported here does not represent the seroprevalence at the time of publication.

Community-level seroprevalence estimates can complement case-based and syndromic surveillance as a tool to understand local transmission and the extent of past infection in a population. The relatively low seroprevalence estimate in this report suggests that most persons in the catchment area had not been infected with SARS-CoV-2 by the end of April. Continued mitigation measures to prevent infection, including social distancing, consistent and correct use of face coverings, and hand hygiene, remain essential to controlling the spread of SARS-CoV-2 in the community.

**Summary**

**What is already known about this topic?**

SARS-CoV-2 infection in persons who are asymptomatic or not tested might not be recognized by case-based and syndromic surveillance; therefore, the population prevalence of past infection might be unknown.

**What is added by this report?**

A community seroprevalence survey, conducted in two counties in metropolitan Atlanta during April 28–May 3, using a two-stage cluster sampling design and serologic testing, estimated that 2.5% of the population had antibodies to SARS-CoV-2.

**What are the implications for public health practice?**

Serologic surveillance can complement case-based and syndromic surveillance. At the time of this survey, most of the two-county population had not been previously infected with SARS-CoV-2, highlighting the importance of continued mitigation measures to prevent infection, including social distancing, consistent and correct use of face coverings, and hand hygiene.

## Footnotes

* An illness was categorized as one compatible with COVID-19 if symptoms met the Council of State and Territorial Epidemiologists (CSTE) clinical criteria in the case definition, including 1) cough, shortness of breath, or difficulty breathing or 2) two or more other symptoms (fever [measured or subjective], chills, rigors, myalgia, headache, sore throat, new olfactory and taste disorders). https://cdn.ymaws.com/www.cste.org/resource/resmgr/2020ps/interim-20-id-01_covid-19.pdf.

† Sample size calculations were performed assuming a seroprevalence of 1%, a margin of error of 0.9%, and a design effect of 1.6 to account for the survey design and intra-cluster correlation.

§ https://www.cdc.gov/nceh/casper/sampling-methodology.htm.

¶ Included circumstances such as 1) only a minor at home or awake; 2) a language barrier (Spanish as the main language in a household was not considered a language barrier because materials were translated into Spanish, and Spanish-speaking interviewers were available); 3) an inaccessible household; and 4) a potential security concern.

** US Department of Health and Human Services, Title 45 Code of Federal Regulations 46, Protection of Human Subjects.

†† This test was authorized by the Food and Drug Administration for emergency use only. Method verification was completed at CDC in a CLIA-certified diagnostic reference laboratory. Test results were automatically calculated on the VITROS Immunodiagnostic System by dividing the Signal for the test sample to Cutoff (S/C). Specimens with S/C <1.0 are interpreted as nonreactive for anti-SARS-CoV-2 total. Specimens with S/C ≥1.0 are interpreted as reactive for anti-SARS-CoV-2 total.

§§ Of 1,675 approached households, 34.4% refused, 37.8% had no response, and 4.0% requested a return visit at another time that was not completed.

¶¶ Samples for 12 participants could not be tested because of insufficient volume or hemolysis; these participants and a resultant three households were excluded (i.e., no household member had a test result).
